# Spinal Muscular Atrophy Type I With False Negative in Newborn Screening: A Case Report

**DOI:** 10.7759/cureus.42382

**Published:** 2023-07-24

**Authors:** Kazuhiko Hashimoto, Mari Yokokawa, Daisuke Yamashita, Kotaro Yuge, Yoshikazu Otsubo

**Affiliations:** 1 Department of Pediatrics, Sasebo City General Hospital, Sasebo, JPN; 2 Department of Pediatrics and Child Health, Kurume University School of Medicine, Kurume, JPN

**Keywords:** mlpa, false-negative, newborn screening, survival motor neuron gene, spinal muscular atrophy

## Abstract

Spinal muscular atrophy (SMA) is an autosomal recessive neuromuscular disorder caused by the deletion or mutation of the survival motor neuron 1 (SMN1) gene. The establishment of effective newborn screening (NBS) for SMA is important for early diagnosis so that treatment can be administered in the pre-symptomatic or early disease stages. Polymerase chain reaction (PCR)-based genetic testing with dried blood spots has been used in NBS to detect the homozygous deletion of exon 7 in SMN1, however, this methodology is not able to detect newborn infants with heterozygous deletions and/or point mutations in SMN1. We report the case of a male infant who was diagnosed with SMA despite the NBS being negative for all conditions including SMA. The patient presented with severe hypotonia and muscle weakness from around 14 days of age. SMA was suspected and sequence analysis of SMN1 and SMN2 was conducted using the multiplex ligation-dependent probe amplification (MLPA) method, which revealed compound heterozygous mutations of SMN1. The patient was diagnosed with SMA and started on modulating agents including gene therapy. His motor function improved slightly with treatment, however, his motor development remained prominently retarded by 5 months of age. This case highlights the importance of investigating SMA as a potential diagnosis even when the NBS result is negative.

## Introduction

Spinal muscular atrophy (SMA) is a lower motor neuron disease characterized by muscle atrophy and progressive muscle weakness owing to the degeneration of the anterior horn cells of the spinal cord, which is inherited in an autosomal recessive manner [1]. SMA is caused by a homozygous deletion or a heterozygous deletion combined with point mutation on the other allele on the survival motor neuron 1 (SMN1) gene on chromosome 5q and consequential lack of the SMN proteins, causing degeneration of lower motor neurons [2]. In Japan, the prevalence of SMA is 1.17 per 100,000 people and the incidence is 0.51 per 100,00 live births [3]. The SMN2 gene, which is almost identical to SMN1 and differs only in five nucleotides, is closely located on the same chromosome and encodes the same protein as SMN1 in principle. However, each SMN2 copy can produce only around 10% of functional SMN protein because a silent transition within exon 7 of the SMN2 gene causes exon skipping from most SMN2 pre-mRNA transcripts, which results in a truncated, non-functional variant instead of full-length protein [4,5]. Therefore, the copy number of SMN2 is an important factor in determining the severity of SMA [5].

In recent years, modulating agents, such as nusinersen, onasemnogene abeparvovec, and risdiplam, have received marketing authorization for SMA treatment from several authorities including the Food and Drug Administration and European Medicines Agency [2]. In Japan, the Japanese Ministry of Health approved these drugs in 2017, 2020, and 2021, respectively [6]. Nusinersen and risdiplam act as splicing modifiers of SMN2 and increase the levels of full-length SMN mRNA and protein, and onasemnogene abeparvovec is an SMN1 gene replacement therapy, which uses a non-replicating adeno-associated virus capsid to efficiently deliver wild-type SMN1 gene to motor neuron cells [2]. These treatments can improve the prognosis and motor function in patients with SMA and they should be administered pre-symptomatically to maximize their therapeutic effects [7]. Therefore, an established system of newborn screening (NBS) for SMA is important for early diagnosis.

NBS for SMA is performed by real-time polymerase chain reaction (PCR) using dried blood spots to detect the homozygous deletion of exon 7 in SMN1; it can detect approximately 95% of newborns with SMA [8]. NBS programs for SMA have commenced in some regions of Japan, including the Nagasaki prefecture, and its effectiveness has already been proven [6]. However, approximately 5% of SMA cases are induced by the deletion of exon 7 on one allele and a deleterious variant on the other allele of SMN1 [8], which cannot be detected by the current NBS measures.

Herein, we report the case of a male infant diagnosed with SMA after developing severe hypotonia and muscle weakness in the neonatal period even though the NBS for SMA was negative.

This article was previously presented as a meeting abstract at the 520th Japan Pediatric Society District Meeting of Fukuoka on March 11, 2023, and the 217th Japan Pediatric Society District Meeting of Nagasaki on April 23, 2023.

## Case presentation

The patient was born at 40 weeks and three days of gestation as the first child of healthy non-consanguineous Japanese parents who had no family history of neurological disorders or perinatal abnormalities. The patient was delivered with Apgar scores (appearance, pulse, grimace, activity, and respiration) of 9/10 at one minute and 10/10 at five minutes, and he did not present muscle weakness just after birth. NBS performed at the maternity clinic after birth was negative for all conditions. The patient and his parents returned home, but from around 14 days of age, the patient presented poor movement in the extremities and had a feeble cry. Muscle weakness was observed at 29 days of age (at the one-month health checkup) by a local doctor and the patient was referred to our hospital for further examinations at 44 days of age.

**Figure 1:**
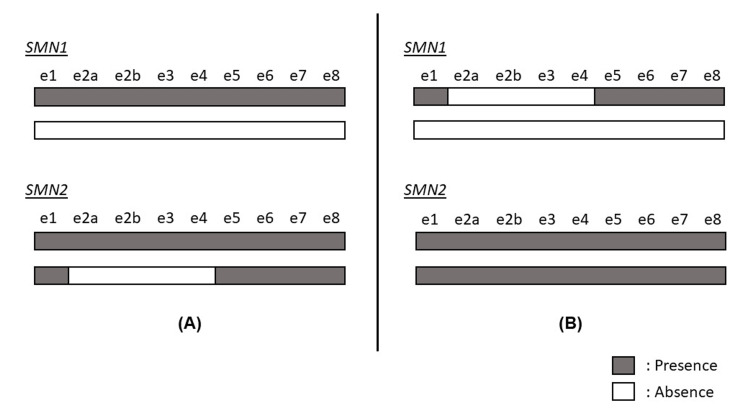
The sequence analysis of the survival motor neuron 1 (SMN1) and SMN2 genes showing two possible scenarios. (A) Heterozygous deletion of SMN1 exons (e) 1–8 and heterozygous deletion of SMN2 e2a–4. With this pattern, the patient is a carrier with one normal copy of SMN1. (B) Heterozygous deletion of SMN1 e1–8 and heterozygous deletion of SMN1 e2a–4. With this pattern, the patient is diagnosed with SMA by compound heterozygous deletions of SMN1 e2a-4.

The patient showed signs of severe hypotonia, such as frog-leg posture, inverted U posture, scarf sign, and heel-to-ear sign at the first visit. He also showed muscle weakness, lacked spontaneous movement, and was unable to perform antigravity movements in both the upper and lower extremities. SMA-related symptoms were also observed, such as paradoxical breathing, tongue fasciculations, and the absence of deep tendon reflexes. Blood test results, including blood cell counts, biochemical analysis, vein blood gas analysis, thyroid function tests, lactate and pyruvate levels, and amino acid analysis, showed no abnormalities. We considered the possibility of SMA based on the general examination, despite the NBS being negative, and thus, we first examined the copy numbers of exons 7 and 8 for both SMN1 and SMN2 using the multiplex ligation-dependent probe amplification (MLPA) method. One copy each of exons 7 and 8 were present for SMN1, while two copies of exons 7 and 8 each for SMN2 were detected. We performed further sequence analysis of SMN1 and SMN2, which revealed that three copies each of exons 1, 7, and 8 were present when considering both SMN1 and SMN2, but only two copies each of exons 2a, 2b, 3, and 4 were present (Figure 1). Structural similarities made it difficult to distinguish between the two genes, and thus, two possible scenarios could be inferred from the available results: a) the heterozygous deletion of SMN1 exons 1-8 and the heterozygous deletion of SMN2 exons 2a-4; or b) the heterozygous deletion of SMN1 exons 1-8 and the heterozygous deletion of SMN1 exons 2a-4. In the first scenario, one copy of normal SMN1 without mutation would be present, while in the latter, compound heterozygous deletions of SMN1 exons 2a-4 would be present, which could lead to the development of SMA.

The patient was thus diagnosed with SMA caused by compound heterozygous mutations and we referred him to a tertiary hospital for gene therapy with onasemnogene abeparvovec. Nusinersen was first administered at 70, 85, and 99 days of age until the onasemnogene abeparvovec was ready. The patient was subsequently administered onasemnogene abeparvovec at 109 days of age. We used the Children's Hospital of Philadelphia Infant Test of Neuromuscular Disorders (CHOP INTEND) score to evaluate motor function. The scores of the CHOP INTEND range from 0 to 64, where the higher the score is, the better motor function. The CHOP INTEND score at the time of the first nusinersen administration was 12/64. At one month after treatment with onasemnogene abeparvovec (140 days of age), the patient performed an antigravity movement, such as elevation of his arm and moving his fingers to his mouth, and his CHOP INTEND score had increased to 20/64; however, his motor development remained delayed. He was admitted to our hospital at 94 days of age owing to respiratory instability with cyanosis, and he regularly required a high-flow nasal cannula as respiratory support. However, he has not required any other respiratory support since his last visit at 168 days of age. Informed consent was obtained from the patient’s parents for this publication.

## Discussion

In our case, SMA was negative by NBS because the patient had compound heterozygous mutations in combination with whole and partial exon deletions on each allele in the SMN1 gene, which could not be detected by this method. Therefore, the patient was not diagnosed with SMA until he presented with symptoms. Clinicians should be aware that patients with these mutations may present as false-negative in NBS.

The severity of SMA is highly variable and the clinical features can be classified into types (0-4) according to the age of onset and maximum motor function achieved, which can be affected by the copy number of SMN2 [9]. Type 0 is the severest form of SMA, in which onset is in utero with reduced or absent movements, contractures, and requirement for mechanical ventilation support at birth, and type 4 is the mildest form of SMA, in which onset is in adulthood and the patient can ambulate independently and they are normal until early adulthood [9,10]. In our case, the patient had two copies each of exons 7 and 8 in the SMN2 gene, which is commonly expected to develop as SMA type 1. Type 1 is further classified into three subtypes by the onset of age: onset within two weeks of age is type 1A, by three months of age is type 1B, and between three and six months of age is type 1C [10]. As the patient presented with severe hypotonia and muscle weakness around two weeks of age, we diagnosed SMA type 1A, which has the most severe prognosis among the type 1 subtypes.

In recent years, preclinical diagnosis of SMA by NBS has attracted attention, and pilot studies have been conducted in many countries around the world [11-13]. In Japan, Shinohara et al. conducted a prospective SMA screening study using dried blood spot samples from 4,157 Japanese newborns, all of which were negative without screening failures [14]. Subsequently, Sawada et al. described an NBS system for detecting the homozygous deletion of exon 7 in SMN1 by real-time PCR and screened 13,587 newborns in a year, identifying one case that was likely to develop SMA. This case was investigated further and the results obtained by the MLPA method revealed that the patient lacked exons 7 and 8 in SMN1 and had three copies of exons 7 and 8 in SMN2; thus, the patient was treated with gene therapy before presenting any symptoms [6].

Although the patient was administered nusinersen and onasemnogene abeparvovec, he showed only a limited improvement in motor function with these treatments. D'Silva et al. reported on 13 children who underwent validated SMA functional motor assessments after treatment with nusinersen and onasemnogene abeparvovec, and nine out of the 13 patients demonstrated an average 7-point increase in CHOP INTEND scores over six months. The majority of children with lower baseline motor function continued to gain meaningful motor skills over the first year following these agents; however, these children continued to have substantial motor impairment [15]. Our patient was treated with these drugs after the onset of SMA symptoms, so his motor development gradually improved but remained delayed.

The false-positive rate of SMA in NBS has been reported in some studies; for example, 252,081 newborn infants were screened in an Australian NBS pilot program, in which the false-positive rate was reported at less than 0.001% [16]. Noguchi et al. reported 10 false-positive cases of SMA among 8,336 newborn infants screened in the Hyogo Prefecture. The authors suggested that the use of heparinized and/or diluted blood in the dried blood spot sample may hamper PCR amplification in SMA-NBS and produce false-positive results [17]. On the other hand, few reports have described the false-negative rates of SMA in NBS. Müller-Felber et al. reported one patient that presented with clinical symptoms of SMA at 68 days of age who screened negative for SMA in the NBS. The subsequent molecular genetic analysis of this patient revealed a combination of a deletion and a point mutation in SMN1 [18]. One of the reasons for the low number of false-negative cases is that the current technique used in NBS detects only the homozygous deletion of exon 7 in SMN1. This implies that false-negative cases are likely to increase in the future because patients with SMA who screen negative in the NBS may later present with SMA-related symptoms. Therefore, it is important for general pediatricians and physical examiners conducting health checkups for infants to be aware of the existence of false-negative SMA cases and the typical symptoms of SMA. In addition, if experts suspect SMA clinically, even when the NBS is negative, they should prioritize sequence analysis of both SMN1 and SMN2 genes, for early diagnosis and treatment.

## Conclusions

In this report, we describe an SMA case with false-negative findings in the NBS. The patient was diagnosed with SMA by sequence analysis of SMN1 and SMN2 after developing SMA-related symptoms. The establishment of effective NBS is extremely important for patients with SMA so that treatment with modulating agents can commence in the pre-symptomatic stage. For now, greater awareness of the potential for false-negative SMA cases in NBS and of SMA-related symptoms will aid in earlier diagnosis and treatment initiation.
